# Trends in academic research on thirdhand smoke using bibliometric analysis

**DOI:** 10.18332/tid/201402

**Published:** 2025-04-03

**Authors:** Joseph K. Ahialey, Yubin Lee, Myung-Bae Park, Jimi Huh

**Affiliations:** 1 Department of Business Administration, Pai Chai University, Daejeon, Republic of Korea; 2 Division of Health Administration, Yonsei University, Gangwon, Wonju, Republic of Korea; 3 Department of Population and Public Health Sciences, Keck School of Medicine of University of Southern California, United States

**Keywords:** thirdhand smoke, secondhand smoke, bibliometric analysis, tobacco, text mining

## Abstract

**INTRODUCTION:**

This study used quantitative analysis to explore the current landscape of thirdhand smoke (THS) research by identifying gaps and emerging trends. Despite growing evidence of health risks associated with THS, research remains sparse, and no public policies address THS exposure. This analysis aimed to inform future studies and policies, in order to mitigate THS-related health risks.

**METHODS:**

Using a bibliometric approach, our literature search identified 227 academic articles and reviews indexed in PubMed between 2009 and 2023. We used Biblioshiny, a bibliometrix R package, VOSviewer, and Excel to analyze the bibliographic data.

**RESULTS:**

Since 2009, an average growth of 14.09% annually has been observed in THS-related publications. Additionally, we found that US institutions are major contributors to THS research. At the country level, studies conducted in the US, China, Spain, Italy, and South Korea are the most prevalent in the THS literature. Our findings indicate that THS research mainly focuses on human participants, health promotion, nicotiana/chemistry, air pollution/indoor analysis, tobacco smoke pollution, adolescent health, odorants/analysis, surface properties, carcinogens, and disease models/animals.

**CONCLUSIONS:**

We analyzed THS research trends and identified the most impactful authors, journals, institutions, and countries. Considering the findings of this study, policymakers should continue policy development and implementation efforts to address THS exposure. The findings of this study can serve as basic reference material for scholars to guide future research directions regarding THS research.

## INTRODUCTION

Research on the harmful effects of tobacco began gaining momentum in the early 1960s in the US^1^. Concerns and awareness regarding the negative impact of smoking on humans led to significant progress in policies that enable people to live in a smoke-free environment. According to a report by the World Health Organization (WHO), as of 2022, over 70% of the world’s population (over 5.6 billion people) is covered by at least one MPOWER measure implemented at the highest level^2^. MPOWER was developed in the WHO Framework Convention on Tobacco Control as a technical package to assist countries in implementing demand reduction^2^. However, the same WHO report noted that over 8 million tobacco-related deaths occur annually. Thus, tobacco use remains one of the biggest threats to public health, making it a global health priority^2^.

The focus on the dangers of tobacco expanded in the 1970s to include the risks associated with secondhand smoke (SHS). Research during this period began to reveal the significant health impacts of SHS, particularly its link to respiratory diseases, cardiovascular issues, and cancer in non-smokers^3^. This led to a surge in studies exploring various aspects of SHS, from its biochemical composition to its effects on various populations. As evidence mounted, public health policies increasingly incorporated measures to reduce SHS exposure, such as smoking bans in public places, workplaces, and multi-unit residences^4^. Over the years, extensive research has been conducted to better understand the mechanisms by which SHS affects health, leading to more stringent regulations and heightened public awareness of the need for smoke-free environments.

In the early 2000s, thirdhand smoke (THS) emerged as a new area of concern in tobacco research. THS refers to the residual nicotine and other chemicals left on indoor surfaces by tobacco smoke. Initial studies indicated that these residues pose significant health risks, especially in children and medically vulnerable individuals^5^. Research on THS has expanded to explore its composition, persistence, and potential health impacts. Studies have shown that THS reacts with common indoor pollutants to form carcinogenic compounds, raising concerns about its long-term effects on indoor air quality and health^6^. Despite growing evidence, THS-related research remains sparse, highlighting the need for further research and public health interventions to address this invisible threat. Similarly, THS, which results from SHS, has increasingly been characterized as a distinct public health risk, particularly for vulnerable pediatric patients who cohabitate with smokers^7,8^. Additionally, evidence suggests that THS exposure is as dangerous as SHS exposure. However, no public policies have been implemented to protect the public against THS exposure^9^. Children are particularly sensitive to THS exposure^5^.

Bibliometrics is a quantitative approach to analyzing academic literature that can provide insights into research trends over time, the impact of specific studies on a given field, and the level of maturity of a scientific field. By utilizing bibliometric analysis, researchers can assess the productivity of authors, influence of publications, and network of citations within a given research area^10^. This method helps identify key areas of focus, emerging trends, and potential gaps in the literature. It is also essential to acknowledge that bibliometric analysis, while predominantly quantitative, often incorporates qualitative insights to enhance interpretation. For instance, keyword co-occurrence and thematic map analyses require qualitative reasoning to contextualize findings and understand emerging research themes. This integrative approach bridges the gap between numerical data and meaningful conclusions, enabling a comprehensive understanding of the field^10^.

To shed light on the difference between previous THS-related reviews and the current study, we briefly discuss the difference between systematic literature review and bibliometric analysis. Extensive research has been conducted on SHS and THS from empirical and experimental perspectives. Furthermore, a growing number of studies have employed systematic literature reviews to explore the existing literature on both SHS and THS^11-14^. The systematic literature review approach focuses on analyzing relatively few studies and is based on qualitative analysis. By contrast, bibliometric analysis focuses on quantitative analysis and explores a larger quantity of documents^10^. For example, Holitzki et al.^14^ employed a systematic literature review approach to analyze 15 documents on SHS and THS marijuana-related literature. Similarly, Díez-Izquierdo et al.^13^ adopted a systematic literature review approach, focusing on 68 articles to update the literature on THS. While THS-related bibliometric studies remain scarce, Tavassoli et al.^15^ most recently explored emerging research trends in SHS and pregnancy-related literature from 1973 to 2020 using bibliometric analysis. Owing to limited bibliometric analyses of THS-related literature, there is a gap concerning the extensive coverage of THS, and the need for comprehensive research on THS remains significant. In contrast to Díez-Izquierdo et al.^13^ and Tavassoli et al.^15^, this study employs a bibliometric approach to analyze a relatively large number of the most recent THS-related articles spanning 2009–2023.

As bibliometric analysis relies on quantitative techniques, it can avoid or alleviate bias that may arise from adopting a systematic literature review^10^. While bibliometric and meta-analyses use quantitative data, bibliometric analysis encapsulates a field’s bibliometric and informational structure by examining the social and structural associations between research components such as authors, institutions, etc., a systematic literature review, in contrast, employs a qualitative approach and is better aligned with confined or niche research areas^10^. By incorporating both approaches, this study enhances the exploration of the current THS literature and underscores the importance of developing public policies to mitigate THS exposure risks. In this study, a bibliometric hybrid approach is employed, integrating quantitative analysis, such as evaluation and interpretation (e.g. co-authorship and keyword analysis^10^, with qualitative analysis to interpret research trends^10^). To date, no studies have conducted THS-related bibliometric analyses. This review explores the present landscape of THS and offers insights into the literature by adopting a bibliometric analysis approach. Further, it highlights the gaps in the current THS literature and underscores the importance of developing public policies to mitigate the risks associated with THS exposure.

Bibliometric analysis is a widely used and rigorous approach for uncovering and examining large volumes of scientific data^10^. It assists in unpacking the evolutionary nuances of a particular field and highlights emerging areas within that field^10^. In the clinical and public health fields, scholars have attempted to collect the results of multiple studies and draw conclusions through systematic reviews or meta-analyses to gain insights into various studies. For instance, Soleimani et al.^11^ examined 159 published articles using a systematic review approach to compare the toxicants produced during each phase of tobacco combustion (mainstream smoke, sidestream smoke, and cigarette butts). They concluded that polycyclic aromatic hydrocarbons and other aromatic hydrocarbons dominated more studies than other compounds^11^. Similarly, Holitzki et al.^14^ analyzed 15 articles to summarize the health impacts of secondhand and thirdhand marijuana smoke and found evidence of a direct association between the tetrahydrocannabinol content of marijuana and passively exposed individuals. Vanzi et al.^12^ investigated eight articles published in EMBASE, CINAHL, the Cochrane Library, and MEDLINE, focusing on the knowledge, beliefs, and behaviors regarding THS among families, caregivers, and parents. They found that 42.4–91% of parents believed THS was harmful, but parental awareness of THS was not always related to the use of smoking bans at home and in cars^12^. Díez-Izquierdo et al.^13^ systematically reviewed 68 articles indexed in the Web of Science (WoS), CINAHL, MEDLINE, and the Cochrane Central Register of Controlled Trials through April 2018. They found 28 articles focused on nicotine concentrations in THS; 21 on exposure and health effects; 16 on beliefs, behaviors, and policies; and 3 on THS in e-cigarettes or hookahs^13^. While these studies employed a systematic literature review approach, each involving a relatively small number of studies and qualitative analysis to synthesize their findings, bibliometric analysis relies on quantitative techniques to explore a larger number of studies^10^.

Thus, we employed a bibliometric approach to analyze THS-related articles published from 2009 to 2023 indexed in PubMed, aiming to reveal the most impactful articles, journals, researchers, and countries, to gain a broader understanding of the work done in THS and to identify historical trends.

## METHODS

### Data extraction

**Figure 1:**
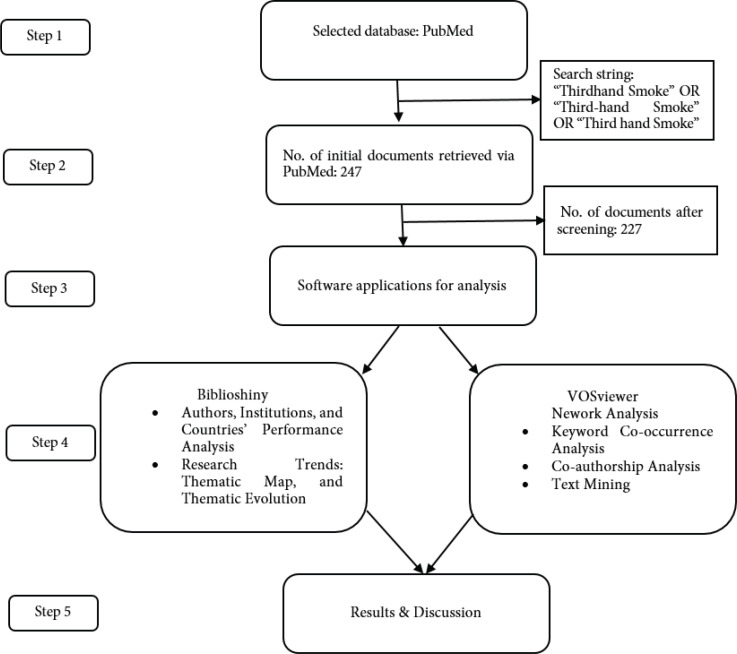
Visual representation of document processing and analysis

The existing literature on bibliometric analysis was primarily obtained from databases such as Scopus, WoS, Lens, and Dimensions. After reviewing the literature on THS, we explored well-known databases such as PubMed, Scopus, and WoS to determine which database had sufficient data for reliable bibliometric analysis, as shown in Figure 1. As PubMed indexed the most THS-related studies among the accessed databases, we selected it as the database from which to extract the documents required for this study. Consequently, as indicated in Figure 1, we set the search strings to ‘Thirdhand Smoke’ OR ‘Third-hand Smoke’ OR ‘Third hand Smoke’ to retrieve all THS-related documents from PubMed. Each database – PubMed, WoS, and Scopus – has its unique strengths and limitations. While PubMed may not be as comprehensive as other databases, it is considered more precise in the field of health and medical sciences^16^. Therefore, we utilized PubMed for the analysis of THS-related documents.

To capture a holistic understanding and flow of THS studies during the sample period, we did not limit the document type to articles and reviews, as this would significantly reduce the number of documents available for reliable analysis. The same applied to the publication year. We initially retrieved 247 articles from PubMed on 30 April 2024. As including documents published in 2024 might affect the yearly average THS literature growth rate, we excluded all articles published in 2024 from the data. Thus, we focused on THS-related literature published in English from 1 January 2009 to 31 December 2023. We chose 2009 as the starting point because no THS-related documents had been registered in PubMed prior to that year. This resulted in 227 articles used for further analysis. Figure 1 shows the step-by-step data processing and analysis used in this review.

### Tools

We used bibliometric analysis to explore the THS literature utilizing Biblioshiny, a bibliometrix R package^17^ which was accessed via R version 4.3.1 and VOSviewer^18^ version 1.6.20 for visualization. Biblioshiny merges the functionality of the bibliometrix package to facilitate the adoption of web apps using the Shiny package environment^19^. The bibliometrix R package offers a collection of tools for quantitative research in bibliometrics and scientometrics^17^.

VOSviewer is a software tool used for creating, visualizing, and exploring bibliometric maps of science^18^. Visualization maps, also known as network visualization in VOSviewer, are threefold: network, overlay, and density visualizations^20^. In this study, we applied network and overlay visualization analyses to investigate the relatedness among terms (network visualization) and trends (overlay visualization) concerning THS research from 2009 to 2023.

Following previous studies, such as that of Wang et al.^21^, we first analyzed the statistics of the terms used, including but not limited to countries, authors, and affiliations. In other words, we summarized the performance of countries, authors, and affiliations. We then examined cooperation among the terms via a network visualization map, such as individual author co-authorship among researchers.

We finalized the analysis by exploring how themes related to THS have evolved over the years through keyword co-occurrence analysis, three-field plots, text mining, thematic maps, and thematic evolution. Conducting these analyses is vital for uncovering research trends and proposing future research directions.

Co-authorship analysis evaluates the interactions among researchers in specific research areas and reveals how scholars interact^10^. Ahmed et al.^22^ stated that co-authorship refers to those who cooperatively work in tandem to produce scientific manuscripts. We employed authors as the unit of analysis in VOSviewer to evaluate how scholars in the THS domain cooperated to develop THS research.

To better understand the trends and themes covered in the THS literature, we analyzed the titles and abstracts of the selected documents by applying VOSviewer’s text-mining functionality to construct an overlay visualization map. This text-mining functionality offers support for constructing term maps using a corpus of documents^23^. The difference between text mining and keyword co-occurrence is that the former is based on titles or abstracts, whereas the latter is based on keywords used in a specific field.

**Figure 2:**
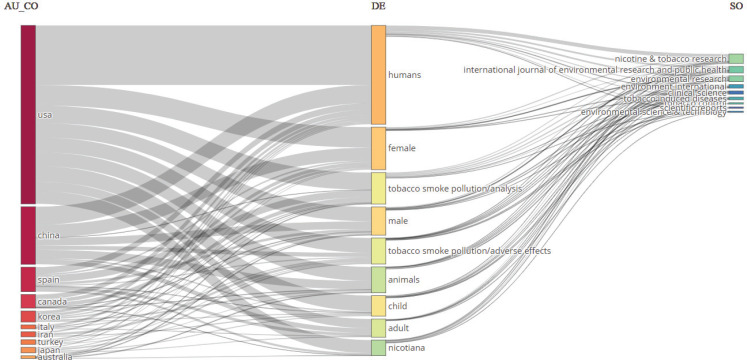
Three-field plot among countries, keywords, and sources regarding THS research

We employed bibliometrix’s three-field plot analysis to determine which countries focused on what topics and in what journals they published related research by analyzing the relationship between countries (left), keywords (middle), and journals (right), as illustrated in Figure 2. This helped identify key countries, popular research topics, and important journals in the THS literature.

A thematic map is a visual tool used in bibliometric analysis to depict themes’ relationships and development status within a particular research domain. It is commonly referred to as a strategic diagram^24-26^ and is constructed based on centrality and density of a theme of Callon et al.^26^.

Thematic evolution involves analyzing the transformation of a research field over consecutive groups of years, known as sub-periods^25^, using a Sankey diagram^27^. For instance, Aria et al.^27^ employed a Sankey diagram to illustrate how various themes were linked and developed in social indicator research over time.

## RESULTS

### Performance analysis

Table 1 presents an overview of the documents used in this review. The publication period spans from 2009 to 2023 with a yearly growth rate of 14.09% in THS-related literature over the sample period. In total, 227 articles from 120 sources authored by 760 researchers were considered for analysis, of which 12 were single-authored documents. Table 1 shows two forms of keywords: keywords plus (ID) and author’s keyword (DE). Keywords plus are words or phrases constructed using a computer algorithm because of their frequent appearance in an article’s reference titles^20^. By contrast, author’s keywords reflect the content of the scientific literature^20^. As indicated in Table 1, both types of keywords registered 756 words each. This result may be partly attributed to the bibliographic database used in this study.

**Table 1:** Descriptive analysis

*Main information about data*	
Timespan (years)	2009–2023
Sources (journals, books, etc.)	120
Documents	227
Annual growth rate (%)	14.09
Document average age (years)	5.91
Average citations per document	0
References	0
**Document contents**	
Keywords plus (ID)	756
Author’s keywords (DE)	756
**Authors**	
Authors	760
Authors of single-authored documents	12
**Authors’ collaboration**	
Single-authored documents	13
Co-authors per document	5.84
International co-authorships (%)	12.33

Document types used in this research are summarized due to space constraints

Similarly, the average number of citations per document and reference was 0. These results were expected because PubMed, unlike other bibliographic databases such as WoS and Scopus, does not include citation- or reference-related metadata. This does not imply that other studies have not cited any of the articles analyzed in this study but, rather, the unavailability of reference and citation-related bibliographic data in PubMed. A mean of 5.84 authors per study was noted during the analysis period. The international collaboration rate among authors was 12.33%.

Supplementary file Figure 1 illustrates the annual scientific output of the THS-related literature from 2009 to 2023. From 2009 to 2016, there was a noticeable increase in the number of annual publications. After that period, the number remained relatively stable without significant fluctuations until 2019. However, in 2020, the publication count sharply declined, followed by a rapid increase again from 2021 onwards. Table 2 highlights the top 10 most influential sources and institutions contributing to THS research. Among the journals, Nicotine and Tobacco Research led with 15 articles, followed by Environmental Research with 11 articles, and both the International Journal of Environmental Research and Public Health and Tobacco Control with 10 articles each. In terms of institutional contributions, most leading institutions are based in the US. San Diego State University ranked first with 132 publications, followed by the University of California (UC) system with 108 publications – dominated by UC San Francisco, along with contributions from UC Riverside, UC San Diego, UC Berkeley, and UC Davis. Lawrence Berkeley National Laboratory secured third place with 70 publications.

**Table 2:** Top ten impactful sources and institutional contributions in THS research (2009–2023)

*Journal*	*Articles*	*Institutional contributions*	*Articles*
Nicotine & Tobacco Research	15	San Diego State University	132
Environmental Research	11	University of California (multiple campuses)*	108
International Journal of Environmental Research and Public Health	10	Lawrence Berkeley National Laboratory	70
Tobacco Control	10	University of Cincinnati	53
Tobacco Induced Disease	9	University of Cincinnati College of Medicine	27
Environment International	7	Universitat Internacional De Catalunya	26
Environmental Science & Technology	7	Nanjing Medical University	22
Clinical Science	5	San Diego State University Research Foundation	20
Scientific Reports	5	Cincinnati Children’s Hospital Medical Center	18
Environmental Health Perspectives	4	Jinan University	16

* UC San Francisco has the overwhelming majority, followed by Riverside, San Diego, Berkeley, and Davis.

Supplementary file Figure 2 offers insight into the productivity of countries regarding THS research based on the corresponding author’s country. The US, China, Spain, Italy, and South Korea are the most impactful countries. The US, China, Spain, and Iran exhibited some level of international cooperation concerning THS research, which is depicted as multi-country publications. By contrast, countries like Italy, Korea, Turkey, and Japan conducted research internally without collaborating with researchers from other jurisdictions, known as single-country publications.

Supplementary file Figure 3 illustrates the influential authors in THS research from 2009 to 2023 based on the number of articles they published. Georg E. Matt (San Diego State University) made the largest contribution with 44 articles, followed by E. Melinda Mahabee-Gittens (University of Cincinnati) with 30 articles, Eunha Hoh (San Diego State University) with 28 articles, and Ashley L. Merianos (University of Cincinnati) with 25 articles.

Supplementary file Figure 4 depicts the cooperation among countries that contribute to the global development of THS. The map comprises lines (links) and colors. The lines and their widths represent the degree of collaboration between countries. The regions on the map^28^ are classified into two main colors: blue and gray. Deep blue shades indicate large scientific production, whereas gray shades denote no publications (Rincón et al.^29^). The US actively contributes to the development of THS literature and has a high level of collaboration with China, as indicated by the size of the link between the two countries. Specifically, the US has co-authored 11 articles with China. The activeness of US researchers corroborates the findings in Supplementary file Figure 4. Furthermore, China collaborated with the UK twice. Finally, the sky-blue-shaded regions show the countries cooperating at least once in publishing THS-related works.

### Network analysis

*Keyword co-occurrence analysis*

The objective of the co-word analysis was to determine the associations between keywords that might be considered most significant at a given point^26^. To analyze the most influential keywords in THS studies, we set the threshold to a default value of 5, which resulted in 79 of the 844 keywords meeting the threshold in VOSviewer. This process generated five clusters, each represented by a different color. Each cluster comprises nodes and links, wherein the nodes depict a term and the links indicating keyword associations.

As shown in Supplementary file Figure 5, the dominant theme in red cluster was tobacco smoke pollution, followed by humans. The relation between these two themes was very close. Examples of other keywords included in this cluster are air pollutants, air pollution, indoor, electronic nicotine delivery system, and environmental tobacco smoke. The green cluster centers on the female theme. Some influential keywords in this cluster were male, adult, thirdhand smoke, and smoking cessation. Other keywords included infant, newborn, family characteristics, smoking prevention, and secondhand smoke.

Furthermore, the blue cluster comprised 14 author’s keywords with nicotine being the most influential keyword. Cotinine and environmental exposure were also impactful in this cluster. The gold cluster, was constructed around animals with peripheral keywords, such as biomarkers, mice, DNA damage, and oxidative stress. As expected, the most influential keyword in this cluster was thirdhand smoke, connected with other noticeable keywords such as child and secondhand smoke. Recently, Merianos et al.^30^ analyzed surface nicotine-derived nitrosamine ketone (NNK) and nicotine in 84 children who lived with smokers and documented that close to half (48.8%) of the children’s home surfaces had noticeable NNK, and 100% had noticeable nicotine. Several studies explored THS in children or infants^7,31,32^.

Supplementary file Figure 6 depicts the trend of author’s keywords over the sample period. Smoking, environmental exposure, smoking cessation, air pollutant, and public health were examples of keywords explored through 2016. From 2017 to 2019, tobacco smoke pollution, humans, female, biomarkers, animals, pregnancy, infant, child, carcinogens, nicotiana, secondhand smoke, and smoke were examined. Some of the keywords that emerged in 2020 were thirdhand smoke, family, cannabis, child health, and smokers.

*Co-authorship analysis: Authors*

The size of a node, as shown in Supplementary file Figure 7, indicates its importance and frequency, and the lines visually represent connections. Researchers are largely divided into three groups. The green cluster represents a significant and frequently occurring group in THS studies with ‘matt, georg e’ (Georg Matt) being the most prominent author. Researchers collaborating with Matt included ‘quintana, penelope j e’ (Penelope J. E. Quintana), ‘mahabee-gittens, e melinda’ (E. Melinda Mahabee-Gittens), and ‘merianos, ashley l’ (Ashley L. Merianos). The red cluster indicates close collaboration among ‘jacob, peyton 3^rd^’ (Peyton Jacob), ‘hang, bo’ (Hang Bo), and ‘mao jian-hua’ (Mao Jian-Hua). The blue cluster is most notably represented by ‘hovel melbourne f’ (Hovell Melbourne F).

*Text-mining analysis*

To fully comprehend the work performed on THS, we used the text-mining feature in VOSviewer to analyze titles and abstracts through overlay visualization. We applied a full counting approach and set the minimum threshold for the terms used in the analysis to 10. This returned 197 terms that met the threshold of 5798 terms. VOSviewer calculated the relevant scores based on these 197 terms, and 60% of the most impactful terms were selected. This resulted in 118 terms used for the analysis. As depicted in Supplementary file Figure 8, terms such as hotel, car, pregnancy, mother, cell, non-smoker, patient, and pediatrician were covered in the THS research in 2016. In 2017, terms such as surface, effect, dust, belief, and smoking ban dominated THS literature. The terms explored between 2018 and 2019 included cigarette smoke, methylnitrosamino, NNA, metabolite, nicotine, concentration, NNK, mouse, liver, and infant. Finally, influential THS-related terms from 2020 onwards included tobacco smoke exposure (including TSE), group, tobacco smoke exposure, behavior, knowledge, cotinine, and hand.

*Three-field plot analysis*

In Figure 2, the leftmost column represents the authors’ country. THS-related research was conducted in the following order, based on the quantity of research: the US, China, Spain, Canada, and South Korea. The middle column lists the keywords. For example, in THS research, the keyword human appeared most frequently, particularly in studies from the US, and was used across all countries, followed by female, tobacco smoke pollution/analysis, and male. The rightmost column indicates the journals in which the keywords were published.

*Thematic map analysis for keyword*

A thematic map is a strategic graph obtained by arranging clusters horizontally depending on the increasing order of centrality and vertically by increasing the order of density^24^. High-centrality themes are located closer to the center of the map, indicating that they have a significant influence and numerous connections to other themes. A theme with high density indicates that the research articles within this theme are closely interrelated, often referencing and building on each other’s work. High density suggests a well-developed, focused, and mature research area.

**Figure 3:**
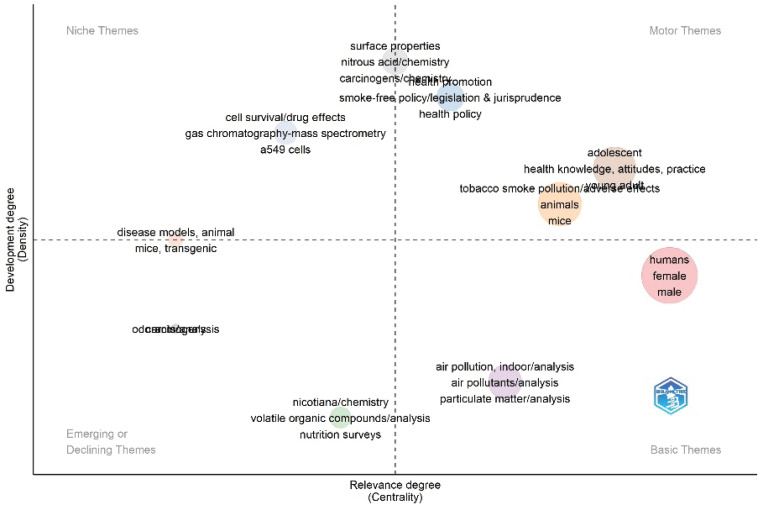
Thematic map of THS research (2009–2023)

Figure 3 illustrates the grouping of researcher keywords into four main quadrants: motor themes, basic themes, emerging or declining themes, and niche themes. The motor-themes quadrant presents well-established themes within a domain. In other words, they demonstrate a high level of development^24^. The well-developed themes represented in the motor-themes quadrant were located in the health promotion, tobacco smoke pollution/adverse effects, and adolescent clusters. As expected, these themes were the lifelines of the THS-related literature. The basic-themes quadrant includes general topics in THS-related literature. The human and air pollution, indoor/analysis clusters were included in this quadrant. The top three themes in the human cluster (Cluster 1) are human, male, and female. Next, the air pollution, indoor/analysis cluster (Cluster 4) influential themes include (but were not limited to) air pollution and particulate matter. Finally, the constituents of Cluster 8 (surface properties cluster), such as surface properties, nitrous acid/chemistry and carcinogens/chemistry, are positioned in the middle of the niche and motor themes, as indicated in Figure 3.

Surface properties are vital in the THS literature. THS generally exists on household surfaces, such as counters, tables, carpeting, wallboard walls, upholstery, couches, bedding, curtains, and smokers’ skin and clothing^9^. It is appropriate to conclude that the surface properties cluster began to move from the niche themes quadrant to occupy a vital position in the THS research, as it is characterized by a high density and a medium degree of centrality, as illustrated in Figure 2. Some themes in this cluster include carcinogens, nicotiana/chemistry, volatile organic compounds/analysis, and nutrition surveys. Niche themes are peripheral and well-established themes that have attracted less attention over time^24^. Gas chromatography–mass spectrometry, cell survival/drug effects, and a549 cells are some of the themes that fell under the niche themes category.

*Thematic evolution analysis for keyword*

**Figure 4:**
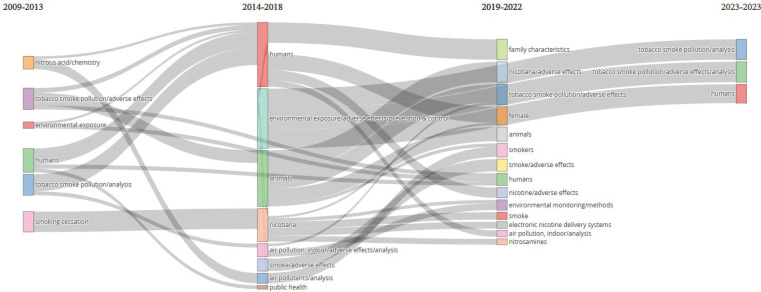
Thematic evolution of THS research (2009–2023)

Figure 4 shows the timeline of the thematic transformation of keywords over four periods: 2009–2013, 2014–2018, 2019–2022, and 2023. It visualizes the relationships between keywords, showing how themes are interconnected and how they evolve. The first phrase explored nitrous acid/chemistry, tobacco smoke pollution/side effects, environmental exposure, humans, tobacco smoke pollution/analysis, and smoking cessation. These evolved into eight topics in the second phase. Examples of these terms are environmental exposure/adverse effects/prevention & control, and public health. In the third phase, these terms developed into 14 terms, including family characteristics, female, electronic nicotine delivery systems, and air pollution, indoor/analysis. Notably, terms such as smoke, adverse effects, and humans dominated the THS-related literature until 2023. This trend is expected to continue.

## DISCUSSION

### Annual scientific output and influential journals

This study revealed several critical points regarding THS research. Interest in THS began to gain momentum in 2009 and continued to fluctuate. First, the steady annual growth rate of 14.09% indicates an increasing academic interest in THS during this period. This was further evidenced by the analysis of 227 peer-reviewed articles, highlighting the extensive and growing research work in this field.

The international collaboration rate among authors was 12.33%, reflecting a moderate level of global interest and cooperative efforts in THS research. This collaboration rate suggests recognition of the global impact of THS exposure and indicates the need for diverse multinational research efforts to comprehensively address this public health issue.

As shown in Supplementary file Figure 1, the annual scientific production steadily fluctuated from 2009 to 2019, significantly decreased in 2020, surged in 2021 and 2022, and sharply declined again in 2023. This trend suggests the need to investigate potential causes, such as budget cuts to funding agencies, shifts in research interests, and external factors, such as the COVID-19 pandemic, which could have impacted research activities.

In early 2020, the publication of COVID-19-related articles in medical journals increased substantially^33^ as many journals streamlined their editorial processes and accelerated publication speed^34^. This led to a decreased volume of other medical and health-related articles unrelated to COVID-19^35^. During the early phase of the pandemic, research on THS may not have progressed owing to various challenges, such as workplace closures and difficulties in collecting data in the field. In 2021, the number of publications increased again, potentially as research slowly returned to normalcy. Global research productivity in the medical and healthcare fields increased during the pandemic with significant growth particularly in the life sciences and biomedical fields. This is because researchers had more time to focus on their work when working from home^36^. However, the decline in THS-related publications in 2023 is more likely to reflect broader trends in journal publishing rather than a diminished interest in THS itself. Notably, from 2020 to 2022, there was a significant surge in publications driven by COVID-19-related research^37^, and the decrease observed in 2023 may indicate a return to pre-pandemic levels. For instance, MDPI reported a 5.9% decrease in the number of publications in 2023 compared to the previous year. However, this trend may be specific to certain publishers, as Elsevier, the largest publisher in this field, reported a slight increase in publication volume in 2023^38,39,^. Therefore, the decline in publications in 2023 requires further investigation to determine whether it resulted from an overall post-pandemic reduction in publications or a specific decline in THS research.

Among the most influential sources, journals such as Nicotine & Tobacco Research, Environmental Research, International Journal of Environmental Research and Public Health, and Tobacco Control stand out. The THS studies were mainly published in tobacco and environmental health journals. Their frequent publications of THS-related studies underscore their pivotal role in disseminating significant research findings and advancing the field.

### Institutional contributions and geographical distribution of research productivity

Our findings reveal that the US dominates THS research with significant contributions from San Diego State University, the UC system, and the Lawrence Berkeley National Laboratory. This concentration of research efforts within the US highlights the country’s leading role in addressing THS issues, possibly because of higher funding availability and greater public health emphasis on tobacco-related studies.

The US, China, Spain, Italy, and South Korea lead in the geographical distribution of research productivity. The high level of international cooperation among the US, China, Spain, and Iran contrasts with the more insular research approaches adopted by Italy, Korea, Turkey, and Japan. This dichotomy suggests varying national strategies and priorities in addressing THS research with some countries leveraging international expertise while others focus on domestic capabilities or research limited to domestic targets.

### Author influence and collaboration

Influential authors in THS research, as illustrated in Supplementary file Figure 3, include Matt, Mahabee-Gittens, Hoh, and Merianos. They are all researchers involved with the US-based Thirdhand Smoke Resource Center (https://thirdhandsmoke.org). The prominence of these researchers reflects their substantial contributions to advancing knowledge of THS and their pivotal roles in shaping research agendas.

The collaborative network underscores the interconnectedness of the global research community with notable collaborations between the US and China enhancing the depth and breadth of THS research through shared expertise and resources.

Network analysis and text mining further elucidated the complex landscape of THS research. Co-authorship analysis revealed key collaborative clusters, highlighting influential researchers and their networks. Network analysis identified two major research groups leading in this field. One group included Matt, Mahabee-Gittens, Quintana, and Merianos, and the other included Peyton Jacob, Bo Hang, and Jian-Hua Mao. These groups are at the forefront of THS research.

### Emerging trends

The keyword co-occurrence analysis and thematic map analysis provided a detailed view of the evolving focus areas within THS research. Dominant themes, such as tobacco smoke pollution, environmental exposure, and public health, indicated a comprehensive approach to understanding the multifaceted impact of THS. Thematic evolution showed a progression from foundational studies on environmental exposure and adverse effects to more nuanced topics, such as family characteristics and electronic nicotine delivery systems. This evolution reflects the field’s maturation and the expanding scope of THS research to include broader environmental and public health contexts. For example, just as research on the harmful effects of conventional cigarette smoke was followed by studies on the harmful effects of e-cigarettes, research on exposure to and effects of THS from e-cigarettes began to emerge in the late-2010s^40-42^. The current study found that, while studies from 2014 to 2018 predominantly used the keyword human, there has been an evolution and refinement of focus since 2019 with an emphasis on keywords such as family characteristics and female. We predict that this shift can be attributed to an increase in publications on THS exposure in domestic settings^43-45^.

Text mining identifies emerging terms and trends, providing insights into shifting research focuses and emerging areas of interest. In the early stages, keywords such as dust, surface, and smoking ban were networked, indicating that research on THS was driven by studies using surface dust as a primary focus^46,47^. Additionally, the presence of keywords like parent, belief, and intervention suggests that there were studies examining parents’ knowledge, perceptions, and intervention programs related to THS^48,49^. Furthermore, even after 2020, keywords like behavior and knowledge remained interconnected, indicating that research on THS continued to be a significant topic^13,48^. The emergence of the word hand alongside tobacco smoke exposure signifies an increase in studies using tobacco smoke exposure as a primary keyword. This reflects the beginning of research on hand nicotine as an exposure marker^49,50^.

### Future directions

It is essential for academic researchers to identify research trends and gaps. The insights gleaned from this comprehensive analysis of the THS literature underscore the dynamic and evolving nature of this field. The observed trends and patterns highlight several critical areas for future research and development.

First, various studies have attributed the recent decline in publication volume, especially after the surge in 2021 and 2022, to external factors, such as the COVID-19 pandemic, which affected THS and other research activities. Therefore, future trends must be observed to determine whether the decline continues or whether there is a rebound, to establish grounds for considering the pandemic period as a temporary anomaly.

Second, the international collaboration rate of 12.33% reflects a moderate level of global interest and cooperation. To comprehensively address the global public health issue of THS exposure, multinational research must be fostered and enhanced. Diverse international collaborations can leverage different expertise and perspectives, leading to more holistic and effective strategies for mitigating THS-related health impacts.

Third, as THS research progresses, it becomes essential to maintain a multidisciplinary approach that integrates environmental science, public health, and policy studies. This multipronged approach is crucial for effectively addressing the multifaceted challenges posed by THS exposure. Encouraging collaboration across these disciplines will facilitate the development of comprehensive interventions and prevention strategies.

Finally, the thematic evolution of THS research indicates a shift towards more nuanced topics such as family characteristics and electronic nicotine delivery systems. Future research should explore these emerging themes, particularly the impact of new technologies and products on THS exposure. Additionally, the use of advanced methodologies such as hand nicotine measurements should be expanded to enhance the accuracy and relevance of exposure assessments.

The findings of the current study highlight the need for continued policy development and implementation efforts to address THS exposure. Policymakers should consider the latest research insights when designing regulations and public health interventions aimed at reducing THS exposure, especially in vulnerable populations such as children and non-smokers. By addressing these critical areas, future research can further enhance our understanding and management of THS exposure, ultimately contributing to improved public health outcomes.

### Limitations

Some limitations of this study should be considered. First, the scope of this study was limited to the past 14 years; therefore, it did not cover studies before and after the sample period. Second, unlike previous bibliometric research, we used PubMed as the only source of academic articles for our analysis. Consequently, articles indexed in other databases, such as WoS, the CINAHL, and MEDLINE, may not have been reflected in this research. Nevertheless, PubMed is specifically designed to cover all aspects of health, medicine, and related fields, making it suitable for research that requires comprehensive data on medical and health topics, including THS studies^45^. Therefore, this review contributes to existing THS literature by demonstrating how THS-related research has evolved over time. Nonetheless, further research is needed to merge THS-related bibliographic data from databases such as WoS and Scopus to explore THS global trends and landscape holistically in the future. Another limitation was that since we focused on only ‘Thirdhand smoke’ as a search strategy, other relevant studies may not have been captured. Thus, further research is needed to combine THS with other influential themes uncovered in our study, such as ‘Tobacco Smoke Pollution’ or ‘Secondhand Smoke’, to examine global trends and landscape in THS research.

## CONCLUSIONS

This study provides a comprehensive overview of THS research trends, highlighting key contributors, influential journals, and geographical distributions. The findings emphasize the importance of global collaboration, multidisciplinary approaches, and advanced methodologies in addressing THS exposure. While the United States remains a dominant player, emerging contributions from countries like China and South Korea signal a growing global interest. The study also identifies evolving research themes, such as the impact of electronic nicotine delivery systems and family characteristics, reflecting the field’s maturation. Despite limitations, such as the reliance on PubMed and a 14-year timeframe, this study serves as a valuable reference for guiding future research and policy development to mitigate the health impacts of THS exposure.

## Supplementary Material



## Data Availability

The data supporting this research are available from the authors on reasonable request.
