# Supplementary material for: Trends in academic research on thirdhand smoke using bibliometric analysis
Source: Tob Induc Dis. 2025 Apr 3;23:10.18332/tid/201402. doi: 10.18332/tid/201402 (PMC11966716; doi:10.18332/tid/201402)
Supplement: Supplementary file 1 [file TID-23-45-s1.pdf]

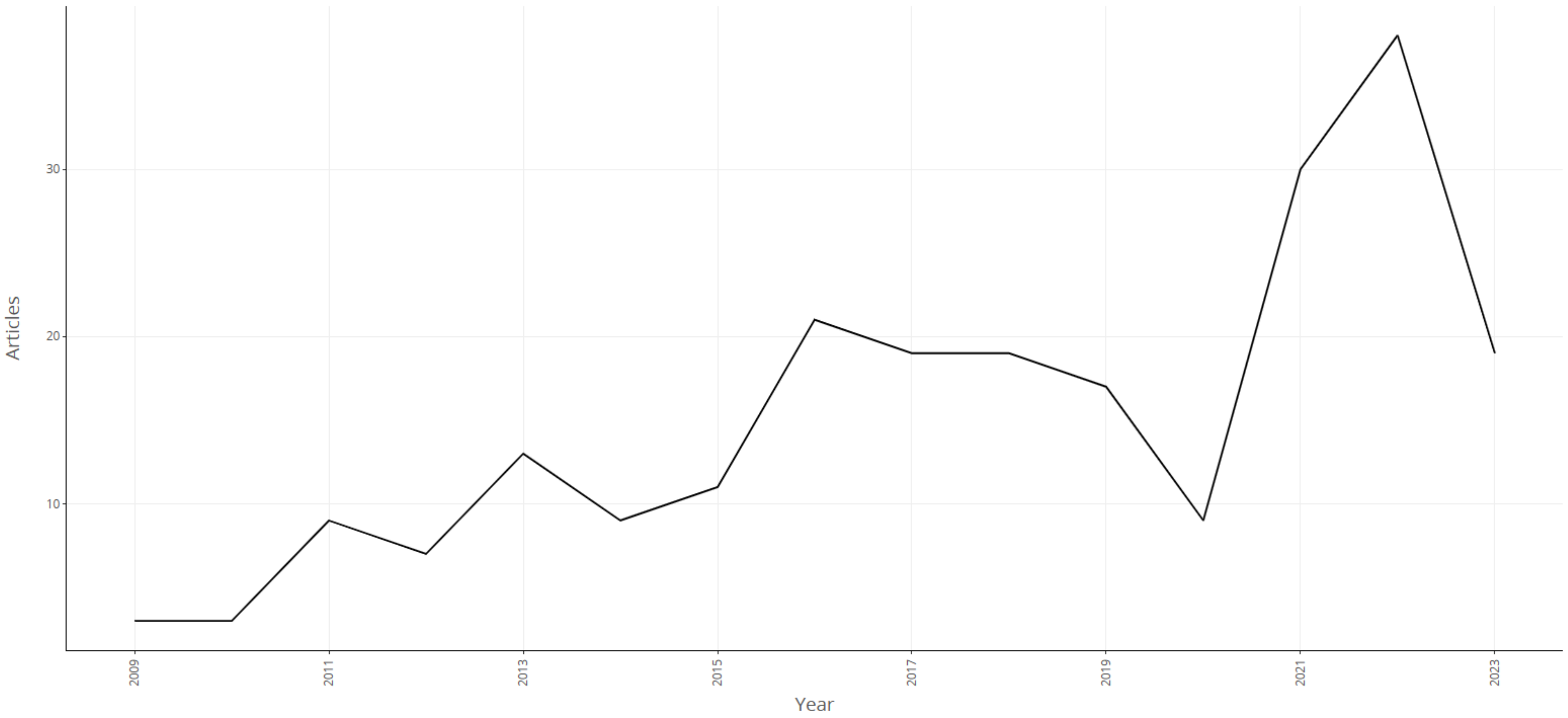

Supplementary Figure 1 Annual Publication of THS-related Literature (2009-2023)

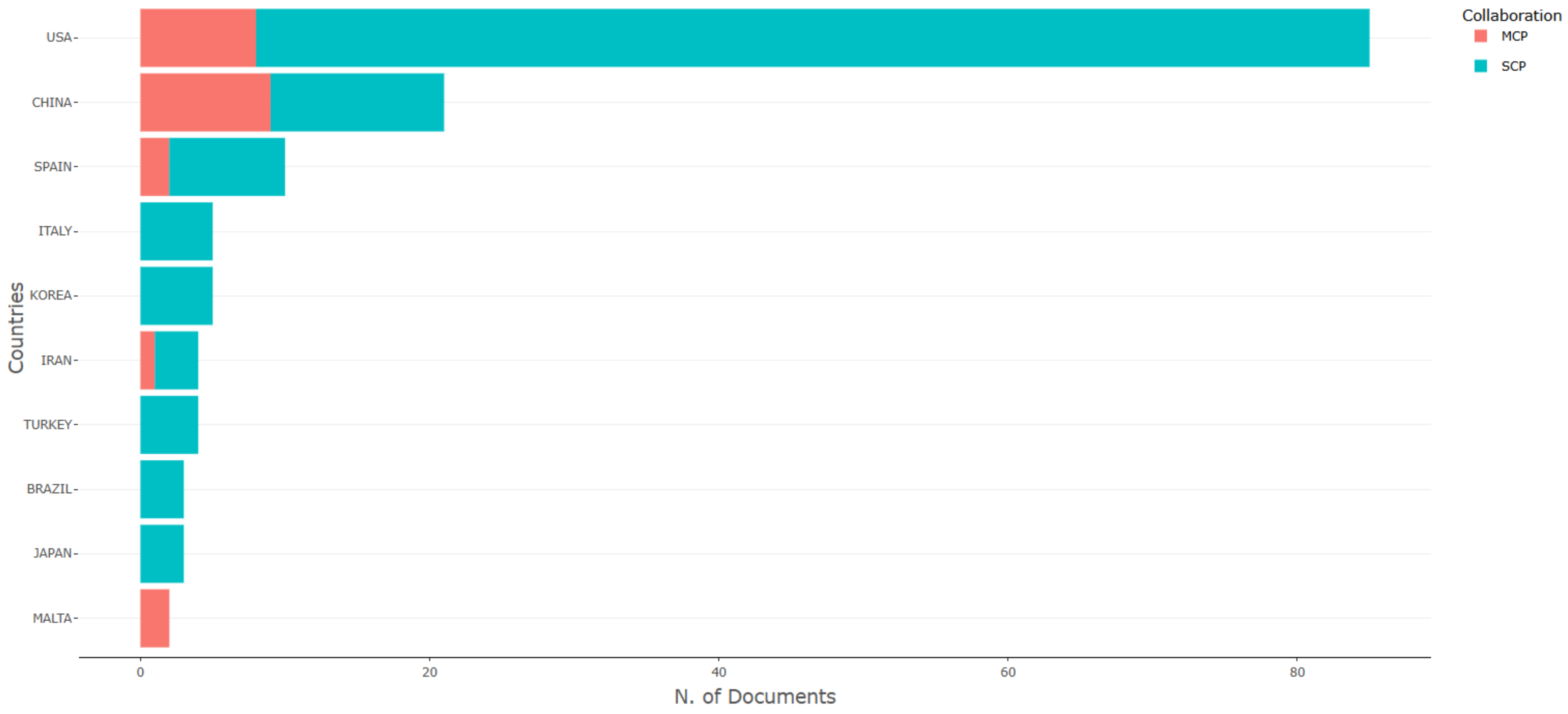

Supplementary Figure 2 Contribution by Country Based on the Corresponding Author's Country (2009–2023)

Note. SCP: single-country publications, MCP: multiple-country publications

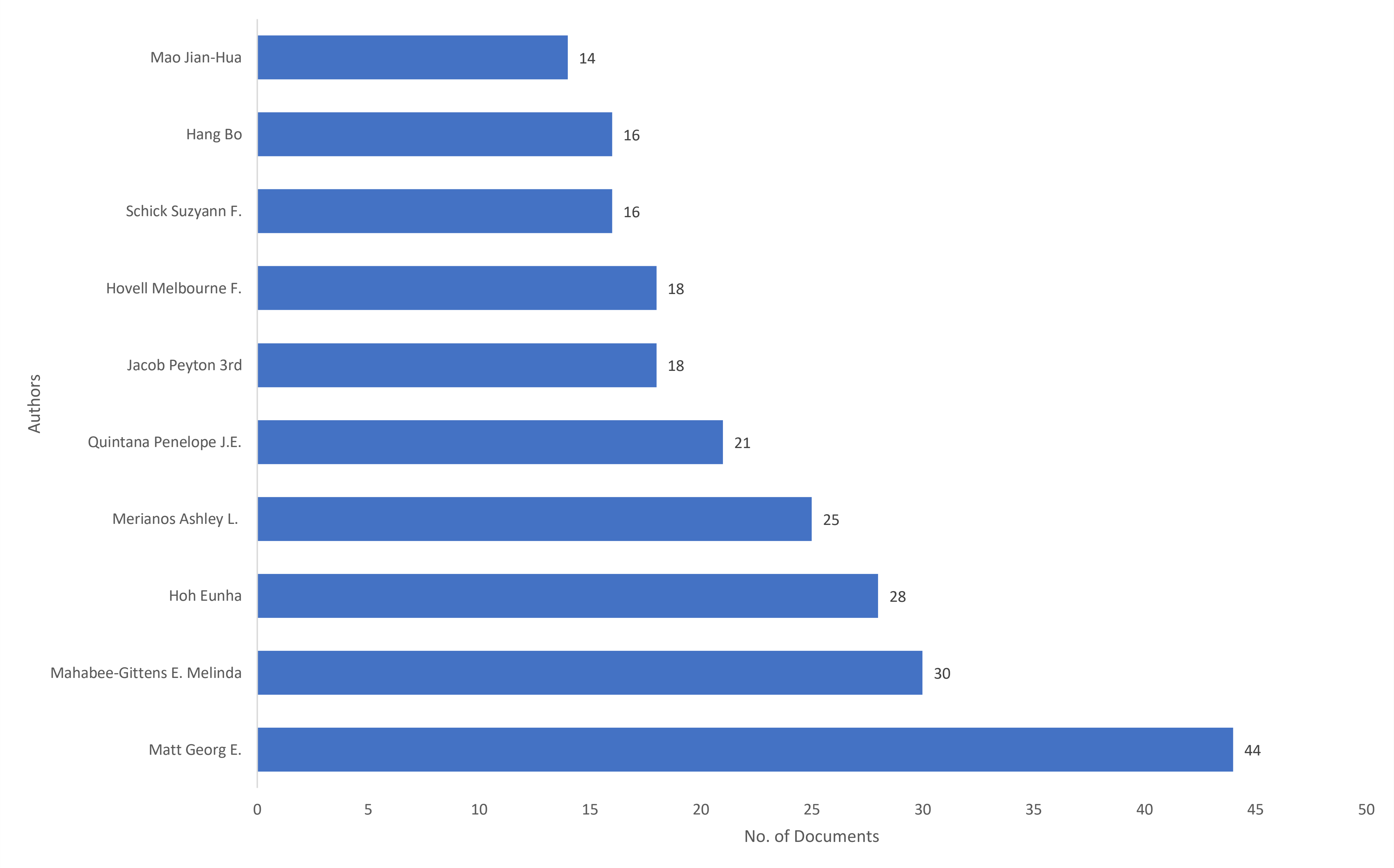

Supplementary Figure 3 Most Relevant Authors in THS Research (2009–2023)

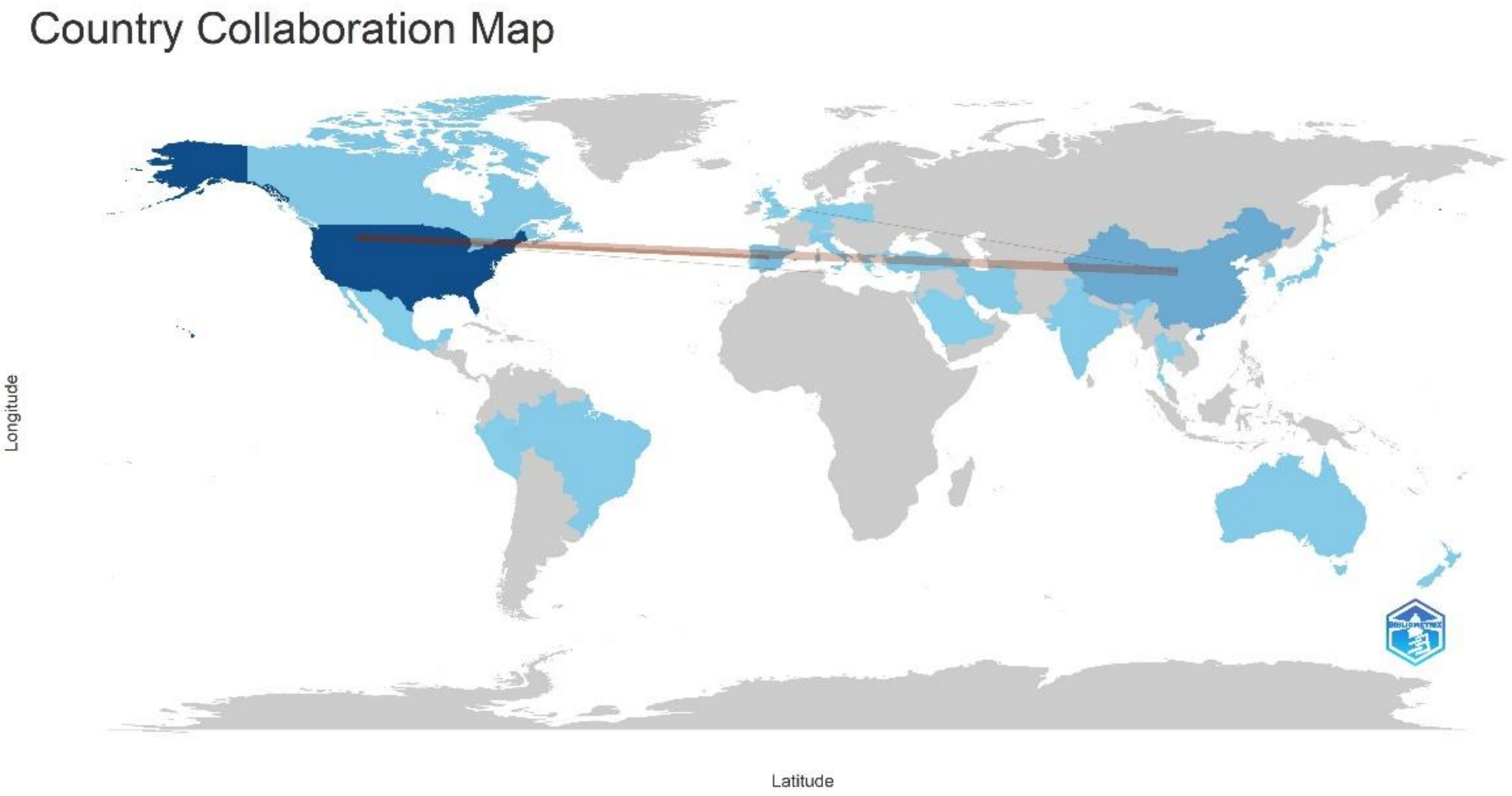

Supplementary Figure 4***:*** **Country Collaboration Map of THS Research (2009–2023)**

This map illustrates the global collaboration in THS research across countries, highlighting the degree of cooperation through links and colors.

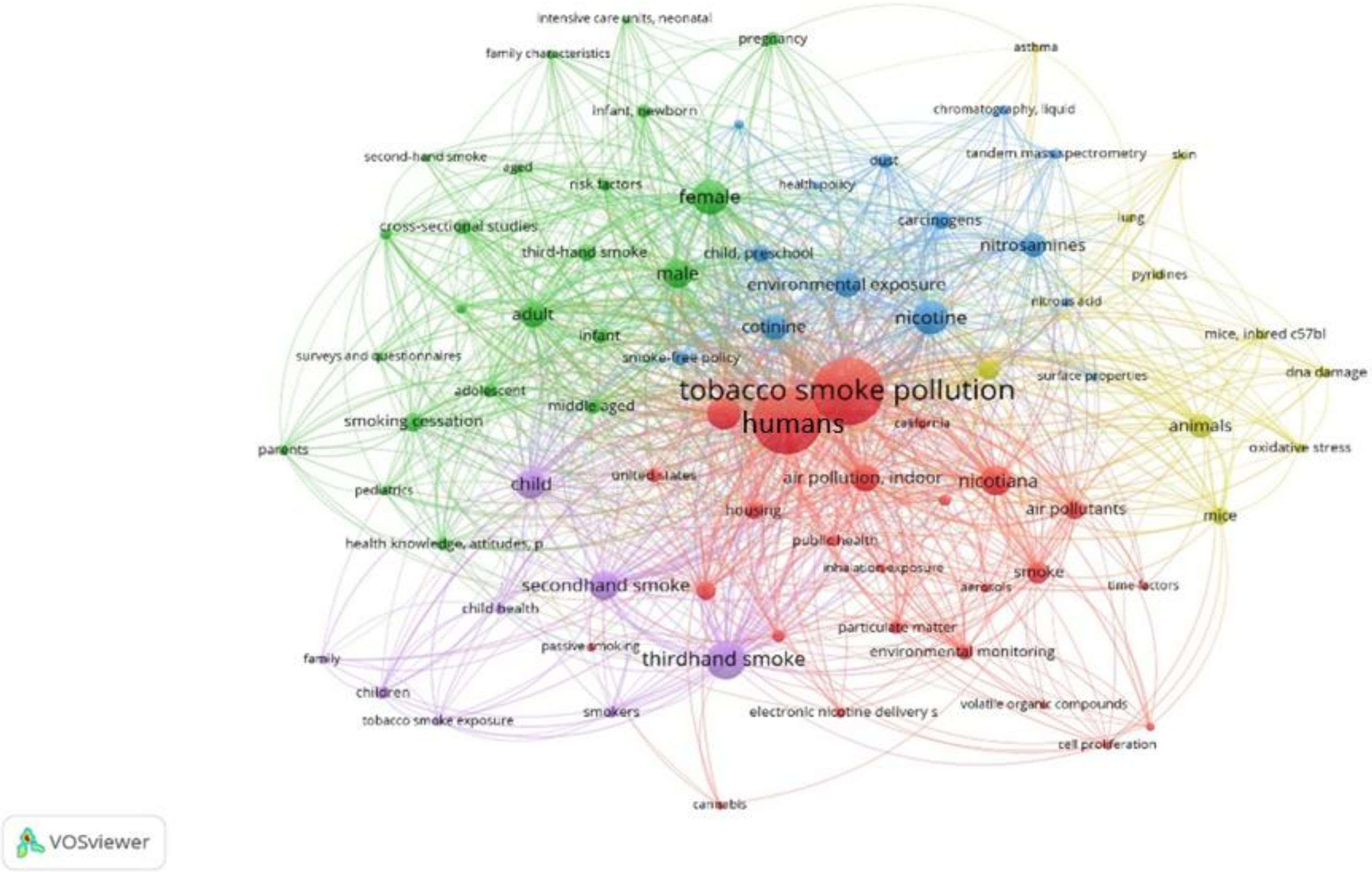

Supplementary Figure 5 Keyword Co-occurrence Network Visualization of THS Research

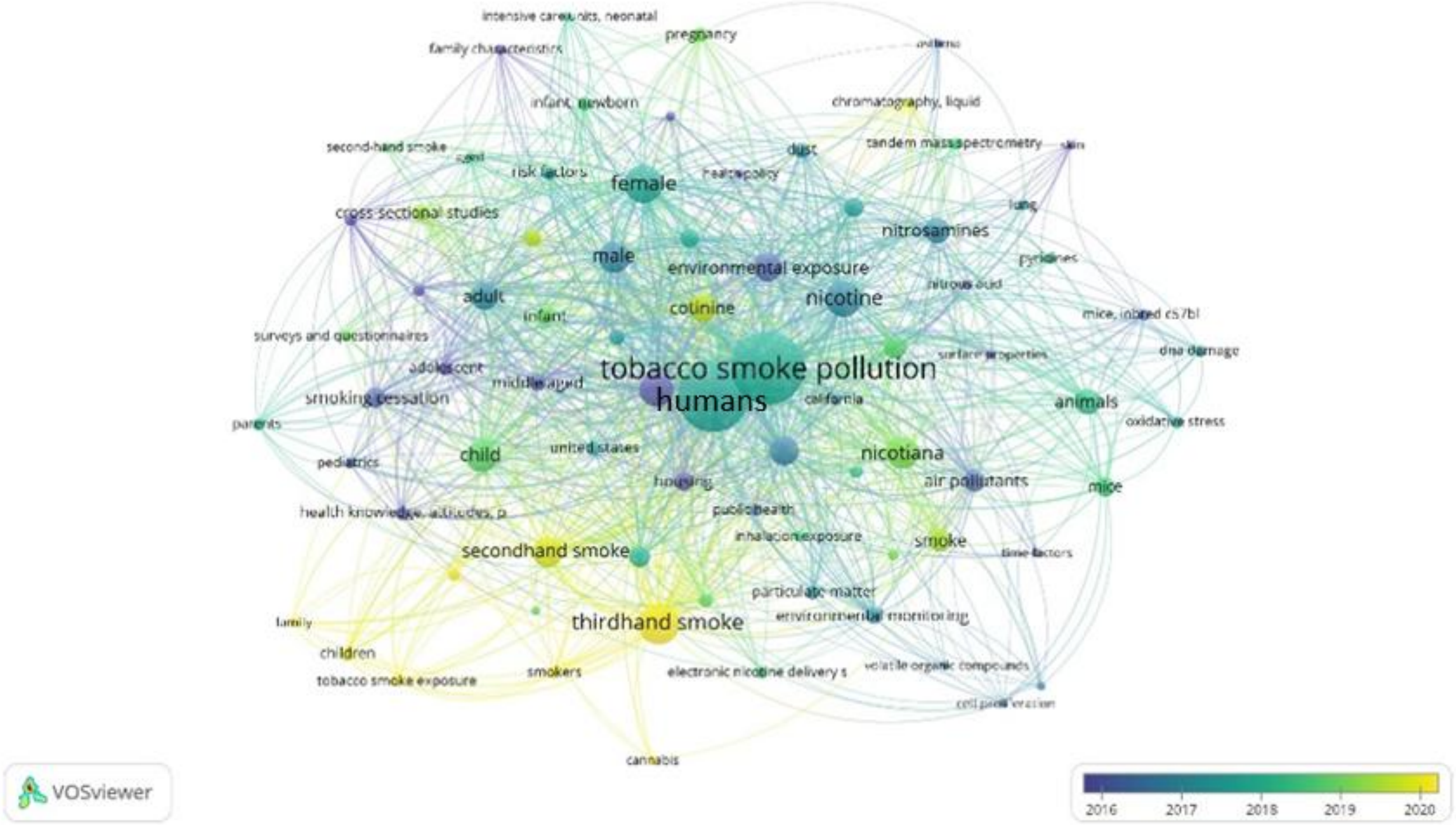

Supplementary Figure 6 Keyword Co-occurrence Overlay Visualization of THS Research

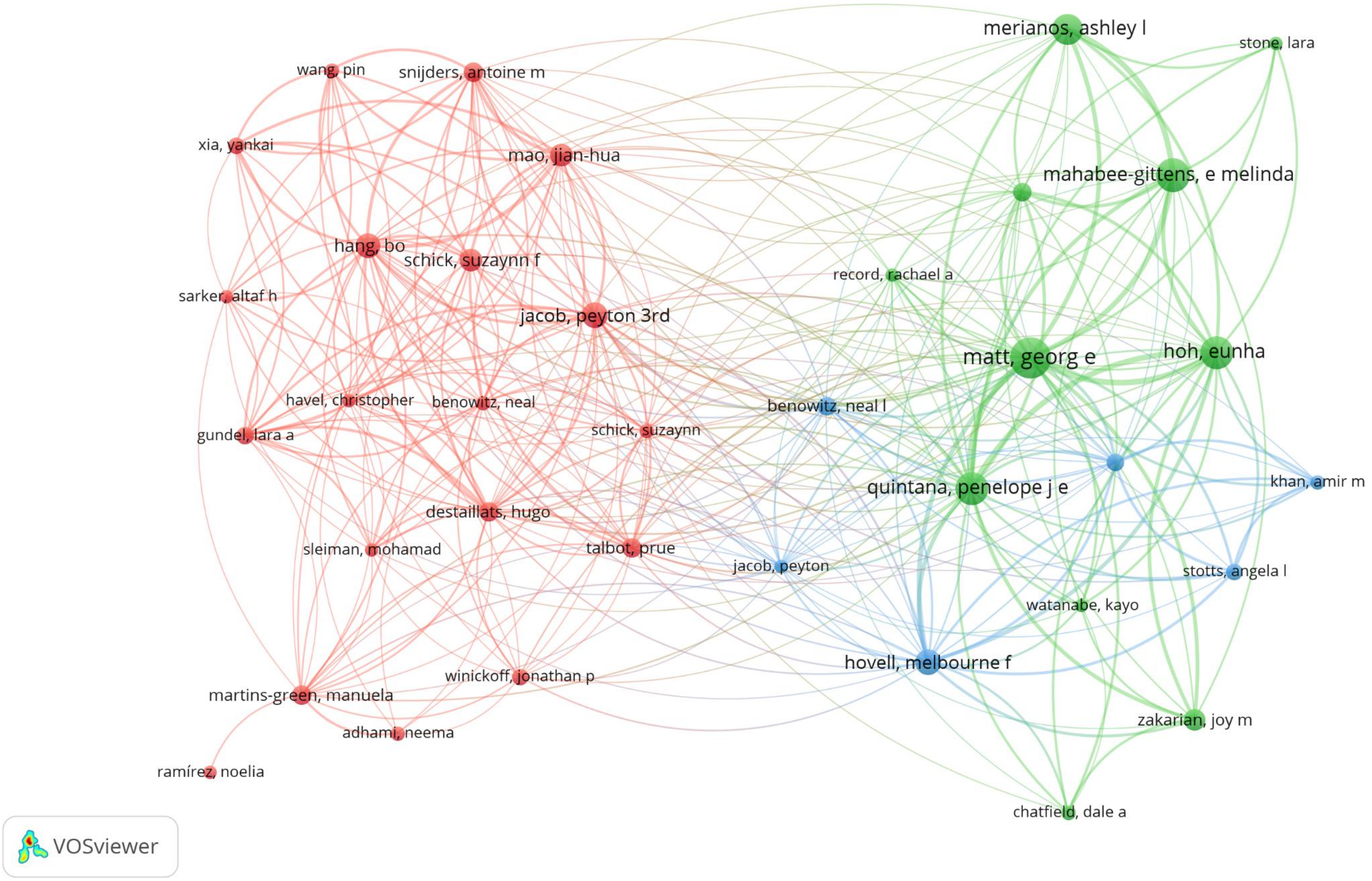

Supplementary Figure 7 Author Co-authorship Network Visualization of THS Research

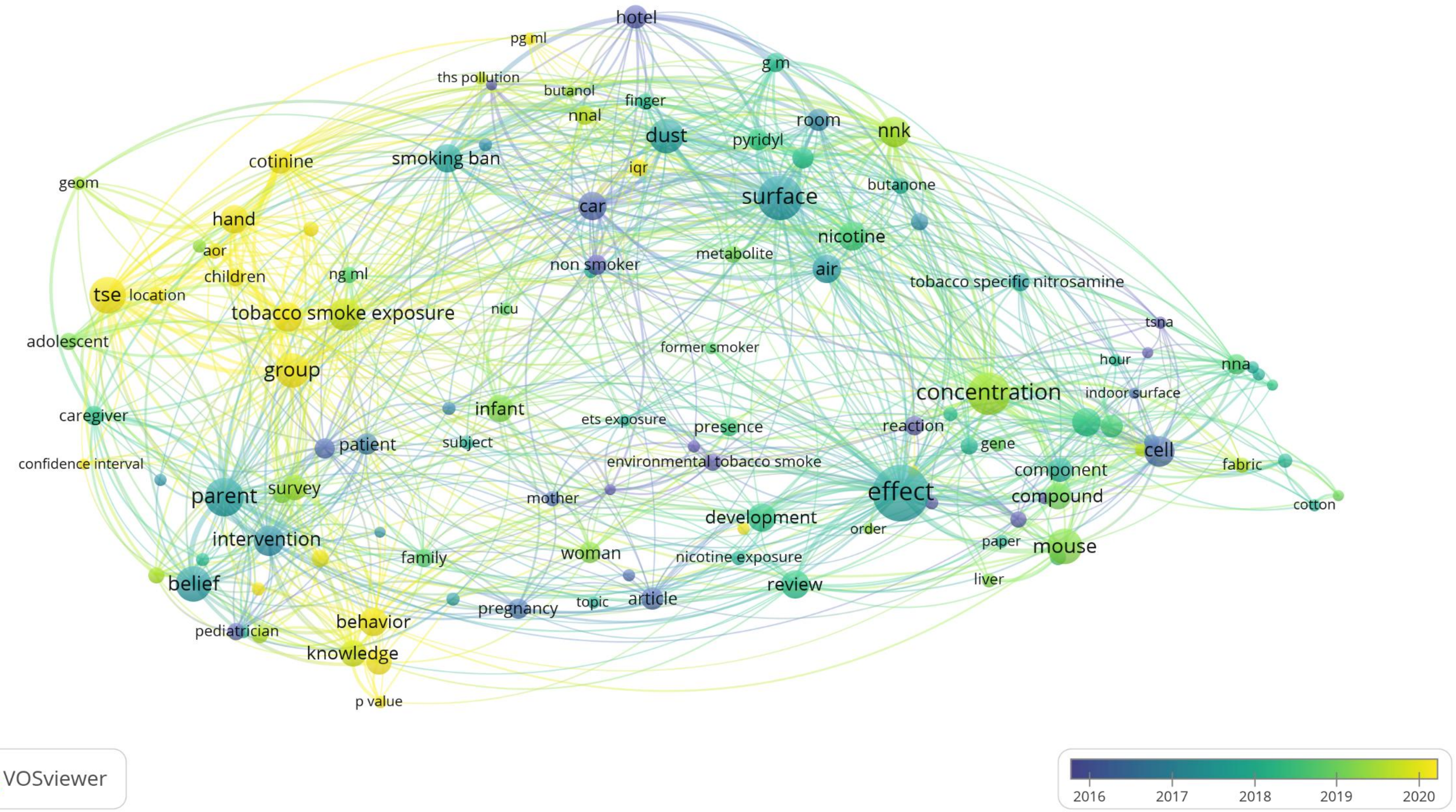

Supplementary Figure 8 Text Mining of THS-Related Literature

© 2025 Kwaku A.J. et al.
